# Cell and animal models of SARS-CoV-2 pathogenesis and immunity

**DOI:** 10.1242/dmm.046581

**Published:** 2020-09-01

**Authors:** Sarah R. Leist, Alexandra Schäfer, David R. Martinez

**Affiliations:** Department of Epidemiology, The University of North Carolina at Chapel Hill, Chapel Hill, NC 27599, USA

**Keywords:** SARS-CoV, SARS-CoV-2, MERS-CoV, Cell models, Animal models

## Abstract

The spread of the novel virus SARS coronavirus 2 (SARS-CoV-2) was explosive, with cases first identified in December 2019, and >22 million people infected and >775,000 deaths as of August 2020. SARS-CoV-2 can cause severe respiratory disease in humans leading to coronavirus disease 2019 (COVID-19). The development of effective clinical interventions, such as antivirals and vaccines that can limit or even prevent the burden and spread of SARS-CoV-2, is a global health priority. Testing of leading antivirals, monoclonal antibody therapies and vaccines against SARS-CoV-2 will require robust animal and cell models of viral pathogenesis. In this Special Article, we discuss the cell-based and animal models of SARS-CoV-2 infection and pathogenesis that have been described as of August 2020. We also outline the outstanding questions for which researchers can leverage animal and cell-based models to improve our understanding of SARS-CoV-2 pathogenesis and protective immunity. Taken together, the refinement of models of SARS-CoV-2 infection will be critical to guide the development of therapeutics and vaccines against SARS-CoV-2 to end the COVID-19 pandemic.

## Abstract (precis)

**Summary:** This Special Article discusses the current model systems to study the novel coronavirus SARS-CoV-2, the pathogen that causes COVID-19.

## Introduction

A novel human coronavirus, SARS coronavirus 2 (SARS-CoV-2), emerged in late 2019 in the city of Wuhan in the Hubei province of China. To date, researchers have identified seven coronaviruses (CoVs) that can cause respiratory disease in humans. These CoVs include OC43, 229E, NL63, HKU1, SARS-CoV, MERS-CoV and SARS-CoV-2. Similar to SARS-CoV and MERS-CoV, SARS-CoV-2 is a betacoronavirus, which can cause severe respiratory disease in humans leading to acute lung disease and death. SARS-CoV-2 quickly spread throughout the world (Zhou et al., 2020; Zhu et al., 2020b), and by January 2020, the World Health Organization (WHO) had declared the SARS-CoV-2 epidemic a global health emergency. By March 2020, the WHO had officially declared the outbreak a pandemic. The SARS-CoV-2 pandemic put a major strain on healthcare systems worldwide, overwhelming hospital intensive care units (ICUs) with coronavirus disease 2019 (COVID-19) patients requiring mechanical ventilation as a result of acute lung injury (ALI) in the form of acute respiratory distress syndrome (ARDS) (Huang et al., 2020; Wang et al., 2020a; Xu et al., 2020; Yang et al., 2020). Some COVID-19 patients can also exhibit organ damage, including acute kidney, cardiac and liver dysfunction (Chen et al., 2020; Wang et al., 2020a; Yang et al., 2020). Comorbidities such as hypertension, cardiovascular disease and diabetes are also associated with more frequent ICU admission and poor outcomes in COVID-19 patients (Wang et al., 2020a).

For the scientific community to combat SARS-CoV-2, the development of infection and pathogenesis models is an urgent requirement. The development of animal models will expedite our understanding of COVID-19 pathogenesis and will enable testing of prophylactic and therapeutic small-molecule SARS-CoV-2 inhibitors, biologics such as SARS-CoV-2-specific monoclonal antibodies, immune modulators such as steroids and interferon therapies, and vaccines. In this Special Article, we discuss the currently available cell and animal models for studying SARS-CoV-2 infection and pathogenesis, and outline how these can be used to improve our understanding of COVID-19 in humans.

## Cell-based approaches to understand the biology, tropism and pathogenesis of SARS-CoV-2

**Fig. 1.**
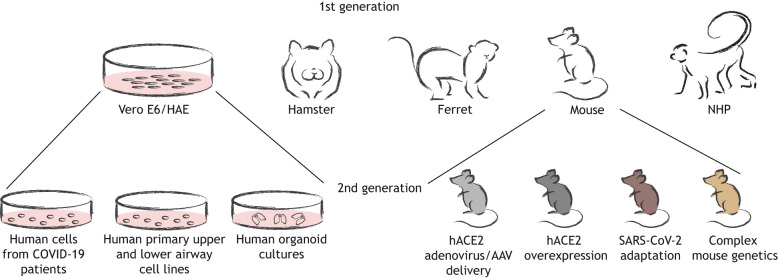
**Cellular and animal models of SARS-CoV-2 pathogenesis.** Cell-based models for SARS-CoV-2 include Vero E6 cells, human ciliated airway epithelial (HAE) cells, human cells from infected patients, human primary upper and lower airway cells, and organoid culture models. Animal models for SARS-CoV-2 include golden Syrian hamsters, ferrets, mice and non-human primates (NHPs). Transgenic hACE2 mice, ACE2 adenovirus- and adeno-associated virus (AAV)-transduced mice and genetically diverse wild-type mice infected with mouse-adapted SARS-CoV-2 constitute a ‘second generation’ of models, which have been optimized based on insight from earlier research.

Cell models are crucial for understanding coronavirus infection biology, growth kinetics and tropism. While human primary lung cell models are highly relevant, common cell lines are also utilized for isolating, growing and screening inhibitors against SARS-CoV-2, and include Vero E6, Calu-3 and A549 cells (Fig. 1) (Hoffmann et al., 2020; Zhou et al., 2020). As with all model systems, immortalized cell lines have advantages and disadvantages. A major limitation of immortalized cell lines (i.e. Vero E6) is that the results obtained, either biological observations or the identification of SARS-CoV-2 inhibitors, must be validated in primary human airway cells or small or large animal models to determine the true relevance of the findings. Although Vero E6 cells express angiotensin I converting enzyme 2 (ACE2), an entry receptor of SARS-CoV-2, they are an immortalized monkey kidney cell line and might not behave as human primary airway cells. For example, in February/March 2020, it was reported that the antimalarial compounds chloroquine and hydroxychloroquine (HCQ) could inhibit SARS-CoV-2 in Vero E6 cells (Liu et al., 2020; Wang et al., 2020b). As a result, several subsequent studies examined the potential efficacy of HCQ against SARS-CoV-2, both in large animal models and in humans. Interestingly, no antiviral effect of HCQ was demonstrated in human airway epithelial cells, and it showed no prophylactic or therapeutic efficacy in hamsters and non-human primates (NHPs) (Maisonnasse et al., 2020; Rosenke et al., 2020 preprint). Moreover, the incidence of the new COVID-19 illness did not differ between participants receiving HCQ when administered as a post-exposure prophylaxis treatment in a double-blind placebo-controlled clinical trial (Boulware et al., 2020). There was no clinical improvement in patients that received HCQ alone or in combination with Azithromycin in a randomized, open-label, three-group controlled trial (Cavalcanti et al., 2020). Together, these studies demonstrate that findings from *in vitro* models should be carefully evaluated and their limitations should be considered when extrapolating results from these models.

Previous studies with SARS-CoV and MERS-CoV demonstrated that specific human airway cells are the targets of coronavirus infection. Similar to SARS-CoV, the entry receptor for SARS-CoV-2 was recently determined to be ACE2 (Letko et al., 2020; Zhou et al., 2020), with co-expression of transmembrane serine protease 2 (TMPRSS2) facilitating infection (Hoffmann et al., 2020). In contrast, MERS-CoV does not use ACE2 but instead enters the cell via dipeptidyl peptidase 4 (DPP4) (Raj et al., 2013). Single-cell RNA sequencing (RNAseq) analysis has shown that lung type II pneumocytes, enterocytes and nasal goblet secretory cells all co-express ACE2 and TMPRSS2 (Ziegler et al., 2020). A separate study defined that human nasal epithelial, large airway epithelial (bronchi and large airway epithelial), lower airway epithelial (bronchiolar and small airway epithelial), and type I and type II pneumocyte (AT1 and AT2) cells are permissive to SARS-CoV-2 infection (Hou et al., 2020). A more recent study of primary human ciliated airway epithelial (HAE) cells found that both ciliated and secretory cells are permissive to SARS-CoV-2 (Zhu et al., 2020a). Interestingly, gut enterocytes were also confirmed to support SARS-CoV-2 replication (Lamers et al., 2020). Similar to airway epithelial cells, the expression of TMPRSS2 and TMPRSS4 on enterocytes promotes SARS-CoV-2 infection (Zang et al., 2020), likely explaining the abdominal pain and diarrhea symptoms in COVID-19 patients (Chen et al., 2020; Wang et al., 2020a). SARS-CoV infects HAE cells and tracheobronchial epithelial cells, but not alveolar epithelial cells (Sims et al., 2005). Both SARS-CoV and SARS-CoV-2 can infect type II pneumocytes (Hou et al., 2020; Mossel et al., 2008). In contrast, MERS-CoV can infect human airway epithelial cell cultures, primary lung fibroblasts, primary lung microvascular endothelial cells and primary alveolar type II pneumocytes (Scobey et al., 2013). Thus, SARS-CoV, SARS-CoV-2 and MERS-CoV all infect type II pneumocytes and human airway epithelial cells, and MERS-CoV has a broader tropism with the capacity to infect primary lung fibroblasts and primary lung microvascular endothelial cells.

Organoid systems are also of interest for modeling SARS-CoV-2 infection and tropism, and for screening SARS-CoV-2 inhibitors. A recent study used a human kidney organoid system to identify clinical-grade soluble ACE2 as an inhibitor of SARS-CoV-2 infection (Monteil et al., 2020), demonstrating the utility of organoids as a physiologically relevant screening platform of SARS-CoV-2 inhibitors. Since the kidney expresses ACE2 and can be infected by SARS-CoV-2 in humans (Bradley et al., 2020), these organoid models will be crucial to understanding the role these cell types play in COVID-19 pathogenesis. Organoid models have advantages over immortalized cell lines, including more closely recapitulating the *in vivo* physiology of the relevant susceptible cell types in the context of an organized tissue. In addition, organoid models can also be used to identify novel SARS-CoV-2-permissive cell types. A recent study of three-dimensional lung organoids identified SCGB1A1^+^ club cells as a novel SARS-CoV-2 infection target (Fig. 1) (Salahudeen et al., 2020 preprint). Although our understanding of severe COVID-19 is rapidly advancing, future studies should consider the use of both upper and lower airway organoid models of SARS-CoV-2 infection, to better understand how infection of these cells modulates immune activation. A limitation of lung, kidney or intestinal organoid models may be the lack of relevant immune components – e.g. macrophages, natural killer cells, eosinophils etc. – that modulate severe COVID-19. Nevertheless, *in vitro* organoid models will be critical to understand the cell biological mechanisms of SARS-CoV-2 infection, and could be a useful tool to screen prophylactic and therapeutic agents in a physiologically relevant system.

Although the target cells of SARS-CoV-2 are beginning to be defined, the role of innate immune responses in these distinct cells are less clear. A recent study that examined the innate immune gene expression landscape in SARS-CoV-2-infected cells found that infection triggers unique inflammatory signatures (Lucas et al., 2020). A separate study corroborated a similar inflammatory cytokine signature profile following SARS-CoV-2 infection of human primary airway and of ferret airway cells, including expression of the genes encoding IL-6, IL-1β, CCL2 and CCL8 (Blanco-Melo et al., 2020). In addition, this study analyzed the transcriptome of bronchial epithelial cells and the serum cytokine and chemokine profiles of deceased COVID-19 patients, and confirmed an increase in inflammatory chemokines like IL-6, IL1RA, CCL2 and CCL8 (Blanco-Melo et al., 2020), suggesting immunologic misfiring in COVID-19. A more recent single-cell RNAseq analysis of human nasopharyngeal and bronchial cells from patients with mild versus severe COVID-19 disease compared to those from healthy patients found an upregulation of genes involved in immune cell and epithelial cell interactions, including *CCL2*, *CCL3*, *CCL20*, *CXCL1* and *IL-1B* (Chua et al., 2020). Taken together, these transcriptomic analyses of human primary cells suggest that COVID-19 leads to a dysregulated immune activation. However, for a complete understanding of these mechanisms, researchers need to complement *in vitro* studies with work in whole-animal models.

## Small rodent and ferret models of SARS-CoV-2

Small animal, particularly rodent, models have been instrumental in our understanding of pathogenic viruses. Golden Syrian hamsters were recently shown to be a suitable animal model that recapitulates certain aspects of SARS-CoV-2 human pathogenesis (Fig. 1) (Imai et al., 2020; Sia et al., 2020). A caveat with this model is that it may be hard to scale given the animals’ lack of broad availability and genetic tractability. Nevertheless, golden Syrian hamsters can be infected with SARS-CoV-2, show clinical signs of disease, can transmit the virus to naïve co-housed animals via aerosols and/or via fomites, and generate neutralizing antibody responses in response to SARS-CoV-2 infection (Sia et al., 2020), underlining the robustness of this animal model with respect to modeling several aspects of human disease. In addition, the golden Syrian hamster model has been used to identify prophylactic modalities against SARS-CoV-2 infection and replication. For example, a recent study demonstrated that passively infused SARS-CoV-2 receptor-binding domain-specific IgG antibodies protected golden Syrian hamsters against lung viral burden, as did passive transfer of convalescent serum to naïve animals (Imai et al., 2020; Rogers et al., 2020). Thus, the golden Syrian hamster should be considered further as a model for COVID-19.

Mouse models have also been instrumental for the evaluation of virally induced immune pathologies, as has been described for SARS-CoV (Bolles et al., 2011). The SARS-CoV-2 Spike protein does not favorably interact with the murine ACE2 receptor, rendering wild-type mice resistant to infection. There are two possible ways to circumvent this problem: adapt the host or adapt the virus. Transgenic knock-in mice, in which the entire murine *Ace2* gene has been replaced with human *ACE2*, are being used to model SARS-CoV-2 pathogenesis (Jiang et al., 2020). Human *ACE2* transgenic (hACE2) mice allow for SARS-CoV-2 replication in the lungs, develop signs of disease and can die from lethal encephalitis (Jiang et al., 2020). Additionally, histopathological analyses revealed infiltration of immune cells into the lungs, resulting in interstitial pneumonia (Bao et al., 2020). Despite recapitulating the immune-mediated lung pathology upon SARS-CoV-2 infection, a disadvantage of the hACE2 transgenic mouse as a model for viral pathogenesis is the lethality caused by neuroinvasion, in addition to the limited availability and relatively slow propagation speed of mice in the setting of a pandemic.

In addition to hACE2 transgenic mice, recent studies described a mouse model that expresses human ACE2 in the lung via adeno-associated virus (AAV) delivery (Fig. 1) (Hassan et al., 2020; Israelow et al., 2020). Although this model is attractive because of its rapid generation and deployment, an important caveat must be considered: the AAV delivery of human ACE2 is not specific for the cells that are permissive to SARS-CoV-2 in humans. Thus, this model may artificially express ACE2 in non-relevant cell types in the mouse airways and lungs (Hassan et al., 2020; Israelow et al., 2020), making pathology and immune response data hard to interpret in the context of human infection of the airways. Nonetheless, as the AAV-ACE2 mouse model can harbor robust viral replication in the lung, it may be suitable to test antibody and drug therapies, which will expedite their human clinical development.

In contrast to golden Syrian hamsters and hACE2 transgenic mice, BALB/c and C57BL/6 mouse strains cannot be readily infected with SARS-CoV-2 human clinical isolates (Dinnon et al., 2020; Imai et al., 2020). Thus, wild-type mice require the modification of the SARS-CoV-2 receptor-binding domain to mediate productive infection and pathogenesis. Predictive modeling and reverse genetic approaches were leveraged to generate a mouse-adapted SARS-CoV-2 (Dinnon et al., 2020). This virus showed efficient replication in BALB/c mice, with a more pronounced disease phenotype, such as weight loss and lung function, in aged mice compared to young mice. This mouse model has also been used to evaluate the prophylactic potential of monoclonal antibodies against SARS-CoV-2 infection, as well as SARS-CoV-2 vaccine efficacy (Fig. 1) (Corbett et al., 2020a; Zost et al., 2020). Inoculating these mice with Venezuelan equine encephalitis virus that expresses the SARS-CoV-2 Spike protein protected against SARS-CoV-2 infection, and neutralizing antibodies were inversely correlated with lung viral titers (Dinnon et al., 2020). Thus, mouse models are suitable for evaluating vaccine performance against viral replication in the lungs. Together, these findings underline the power of small, rodent animal models in improving our understanding of COVID-19 pathogenesis, facilitating the *in vivo* screening of therapeutics and vaccine development.

Ferrets are another animal model that recapitulates several aspects of human COVID-19, including viral shedding and replication from saliva, nasal tissues, lung tissues and intestinal tissues, leading to viral RNA detection in fecal waste (Fig. 1) (Kim et al., 2020; Richard et al., 2020; Shi et al., 2020). Both Kim et al. and Richard et al. reported SARS-CoV-2 transmission from infected to naïve ferrets in co-housed animals and via indirect contact, suggesting transmission via saliva droplet dissemination in close contact or potentially airborne transmission (Kim et al., 2020; Richard et al., 2020). Given that ferrets can support both direct and indirect transmission of SARS-CoV-2, they may be a suitable model for understanding COVID-19 transmission dynamics, which could be important for understanding the role of specific mutations, such as the D614 versus G614 Spike mutations, in the pathogenesis and transmission of SARS-CoV-2 (Korber et al., 2020).

## NHP models of SARS-CoV, MERS-CoV and SARS-CoV-2

So far, 14 mammal species have been investigated for their ACE2 receptor homology to that of humans, with rhesus macaques being the closest match (Zhao et al., 2020). NHP models of viral diseases are thought to be the gold standard for modeling human pathogenesis and for testing clinical interventions because of their anatomic and genetic similarity to humans (Fig. 1). NHPs have previously been used to model SARS-CoV and MERS-CoV pathogenesis. Following the SARS-CoV outbreak in 2003, the rhesus macaque model was shown to exhibit certain degrees of lung pathology lesions similar to those observed in human infection with SARS-CoV (Kuiken et al., 2003), underlining the suitability for NHP models of highly pathogenic CoV infection. Similarly, a pathogenesis study of the wild-type MERS-CoV strain in macaques confirmed seroconversion, the formation of lung lesions and the infection of type 2 pneumocytes, although no renal disease was observed (Yao et al., 2014). In contrast, a rhesus macaque model of MERS-CoV infection with a MERS-CoV infectious clone showed no change in clinical signs such as oxygen exchange, body temperature, complete blood counts or serum chemistry analytes, and no lung lesions (Cockrell et al., 2018). Differences in the results from these two NHP studies could be due to different MERS-CoV challenge viruses. Nevertheless, these studies demonstrate the utility of NHP models as models for human MERS-CoV infections, as they can harbor infection and can exhibit some clinical disease.

Similar to SARS-CoV and MERS-CoV, several NHP models have been developed for SARS-CoV-2 to recapitulate human pathogenesis. One of these is the crab-eating macaque (*Macaca fascicularis*) (Lu et al., 2020; Rockx et al., 2020). Crab-eating macaques exhibit pathological changes in the lung, such as diffuse alveolar damage that coincides with the colocalization of SARS-CoV-2 antigen expression, pulmonary punctate hemorrhage and lung lesions visible by both serial computer tomography and positron emission tomography techniques (Finch et al., 2020 preprint; Lu et al., 2020; Rockx et al., 2020). Likewise, African green monkeys (*Chlorocebus sabaeus*) were recently shown to be a suitable non-human primate model for SARS-CoV-2 (Woolsey et al., 2020 preprint). Upon infection, African green monkeys develop lung lesions, histologic features of pneumonia. Similar to humans, respiratory epithelial cells and type II pneumocytes are the SARS-CoV-2 target cells in this NHP model. Infected African green monkeys also exhibit detectable viral RNA as well as infectious virus in nasal secretions and saliva (Woolsey et al., 2020), suggesting that this model recapitulates some aspects of human COVID-19.

A comparison study of SARS-CoV-2 infection in three different NHP species – rhesus macaques (*Macaca mulatta)*, crab-eating macaques and common marmosets (*Callithrix jacchus*) – showed that 12/12 rhesus macaques had an increase in body temperature, whereas only 2/6 crab-eating macaques and 0/6 common marmosets had an increase in body temperature (Lu et al., 2020). Viral genomes were detected in nasal, throat and anal swabs in all three species. Finally, lung lesions and other histopathological abnormalities were observed in the lungs of these three distinct NHP models (Lu et al., 2020), with both the rhesus and crab-eating macaque exhibiting more pronounced lung pathology. From the NHP models described by Lu et al., the rhesus and crab-eating macaque models mimic some aspects of human COVID-19, shed infectious virus, and appear to be better pathogenesis models compared to the common marmoset (Lu et al., 2020).

The rhesus macaque model has also been utilized to evaluate the therapeutic efficacy of Remdesivir (Williamson et al., 2020), which has been shown to have clinical efficacy in humans (Beigel et al., 2020). Despite the broad utility of NHP models for evaluating SARS-CoV, MERS-CoV and SARS-CoV-2 pathogenesis, the protective efficacy of vaccines and the therapeutic efficacy of antivirals, it should be noted that NHP models, like any model, have some limitations. All NHP models developed to date exhibit less severe disease pathology following infection compared to that observed in humans (Chandrashekar et al., 2020; Cockrell et al., 2018; Lawler et al., 2006). NHPs do not develop the acute lung injury that is observed in mouse models for other related highly pathogenic CoVs, SARS-CoV and MERS-CoV (Cockrell et al., 2016; Roberts et al., 2007). Thus, NHP models may be more appropriate for modeling CoV replication, as they mildly mimic some aspects of human clinical disease. In addition, NHP models are the gold standard in pre-clinical animal testing prior to the initiation of Phase I human clinical trials.

An early question in the field was whether or not SARS-CoV-2 natural immunity could protect against a subsequent infection in humans. Two recent studies demonstrated that SARS-CoV-2-specific neutralizing antibody responses elicited after a primary infection can protect against subsequent re-infection in the rhesus macaque model (Chandrashekar et al., 2020; Deng et al., 2020). Importantly, rhesus macaque models have also been used to demonstrate that a DNA vaccine expressing different forms of the SARS-CoV-2 Spike antigen can elicit protective neutralizing antibodies that correlate with protection against SARS-CoV-2 infection (Yu et al., 2020). This observation has important implications for vaccine development as it suggests that developing a SARS-CoV-2 vaccine may be possible. More recent follow-up studies found that a chimpanzee adenovirus-based (ChAdOx-1) vaccine, currently in clinical trials sponsored by AstraZeneca, protected rhesus macaques from SARS-CoV-2 replication in the lungs (van Doremalen et al., 2020). Moreover, the Moderna mRNA-1273 vaccine, which is now undergoing testing in humans in Phase III clinical trials, protected against both lower and upper airway epithelial cell SARS-CoV-2 replication in rhesus macaques (Corbett et al., 2020b). Similarly, a single shot of an adenovirus (Ad26) vaccine expressing the SARS-CoV-2 Spike protein mediated protection against lower and upper airway epithelial cell replication of SARS-CoV-2, and neutralizing antibody titers correlated with protection in rhesus macaques (Mercado et al., 2020). This Ad26-based vaccine, sponsored by Johnson & Johnson, is also in clinical trials and is set to begin Phase III trials in September 2020. Taken together, these studies suggest that the development of an efficacious SARS-CoV-2 vaccine that can prevent disease in humans can be achievable. In summary, the rhesus macaque most closely recapitulates the SARS-CoV-2 infection observed in humans (Munster et al., 2020; Rockx et al., 2020) and is a useful model to evaluate vaccine efficacy.

## Conclusions

Moving forward, it will be important to improve current animal and cell-based models for a better understanding of SARS-CoV-2 pathogenesis. As clinical studies have reported SARS-CoV-2 shedding from several mucosal tissues, including the mouth, nose, and upper and lower airway epithelium, the development of *in vitro* models, primary cells or organoids, to study SARS-CoV-2 pathogenesis will be crucial. For the refinement of animal models, it will be of highest priority to have ALI animal models that exhibit ARDS. Furthermore, addressing the differences in factors known to influence disease outcome in other viral diseases – such as age, sex, comorbidities and host genetics – will be of utmost importance. The Collaborative Cross reference population of inbred mice has been highly valuable for the development of new animal models (Leist et al., 2016) and additionally reflects the genetic diversity present in the human population. Genetically outbred animal models will be crucial to rigorously evaluate SARS-CoV-2 antibodies, drugs, therapeutics and vaccines to obtain a definitive view of how host genetic variation affects the performance of treatments. Ultimately, combining knowledge from multiple model systems with data from human patients will be key to mitigating COVID-19 and limiting the pandemic.
